# Supplementary material for: Researching New Drug Combinations with Senolytic Activity Using Senescent Human Lung Fibroblasts MRC-5 Cell Line
Source: Pharmaceuticals (Basel). 2024 Jan 4;17(1):70. doi: 10.3390/ph17010070 (PMC10818379; doi:10.3390/ph17010070)
Supplement: Supplementary file 1 [file pharmaceuticals-17-00070-s001.zip › pharmaceuticals-2666130-supplementary.pdf]

Supplementary material

Article: Researching new drug combinations with senolytic activity using senescent human lung fibroblasts MRC-5 cell line.

Authors: Maria Carolina Ximenes de Godoy, Juliana Alves Macedo and Alessandra Gambero.

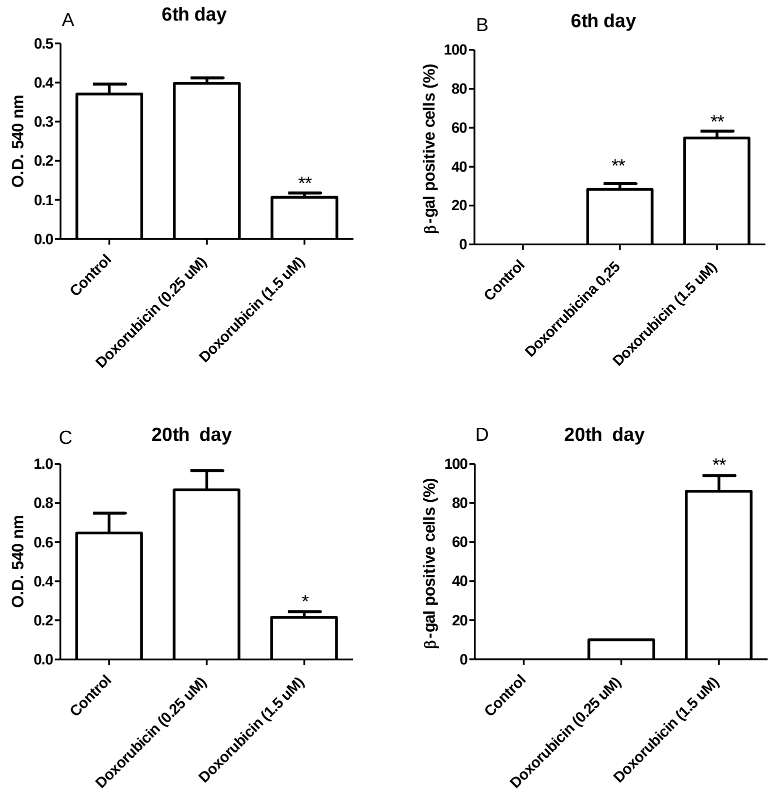

Figure S1: Proliferation and β-gal positive MRC-5 cells after 6 and 20 days of 24 h treatment with doxorubicin 0.25 and 1.5 μM). The experiment was carried out in triplicate with one repetition. * $p<0.05$ when compared to non-treated MRC-5 cells and ** $p<0.01$ when compared to non-treated MRC-5 cells

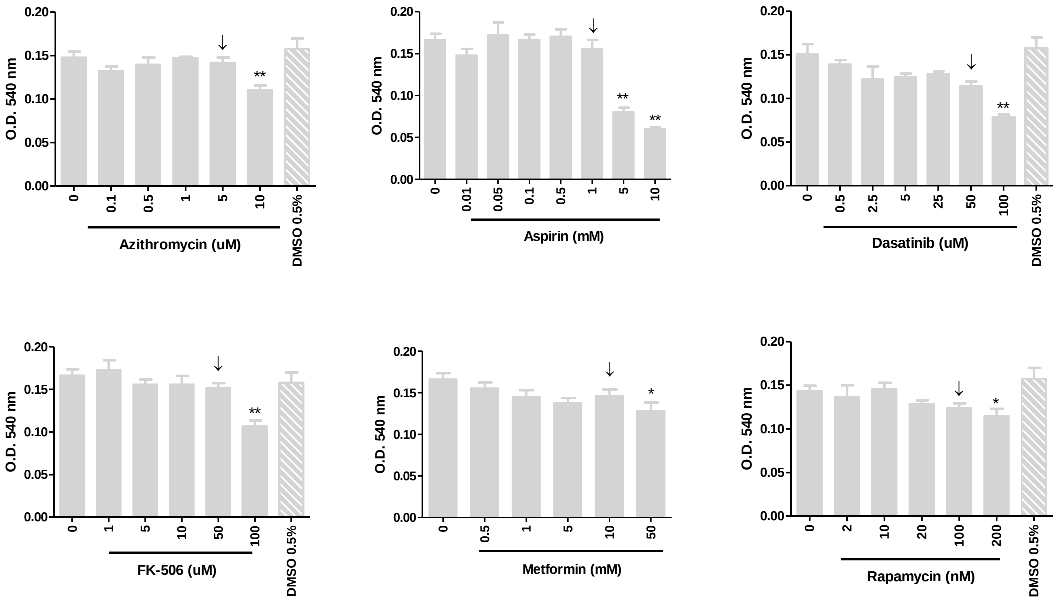

Figure S2: Cytotoxicity evaluated in control MRC-5 cells incubated during 24 h with a concentration range of the drugs Azithromycin (0.1-10 µM), Aspirin (0.01-10 mM), Dasatinib (0.5-100 µM), FK-506 (1-100 µM), Metformin (0.5-50 mM) and Rapamycin (2-200 nM). The experiment was carried out in triplicate with one repetition. * $p<0.05$ and ** $p<0.01$ when compared to non-treated MRC-5 cells. The arrows show the concentration chosen for each drug for subsequent assays.

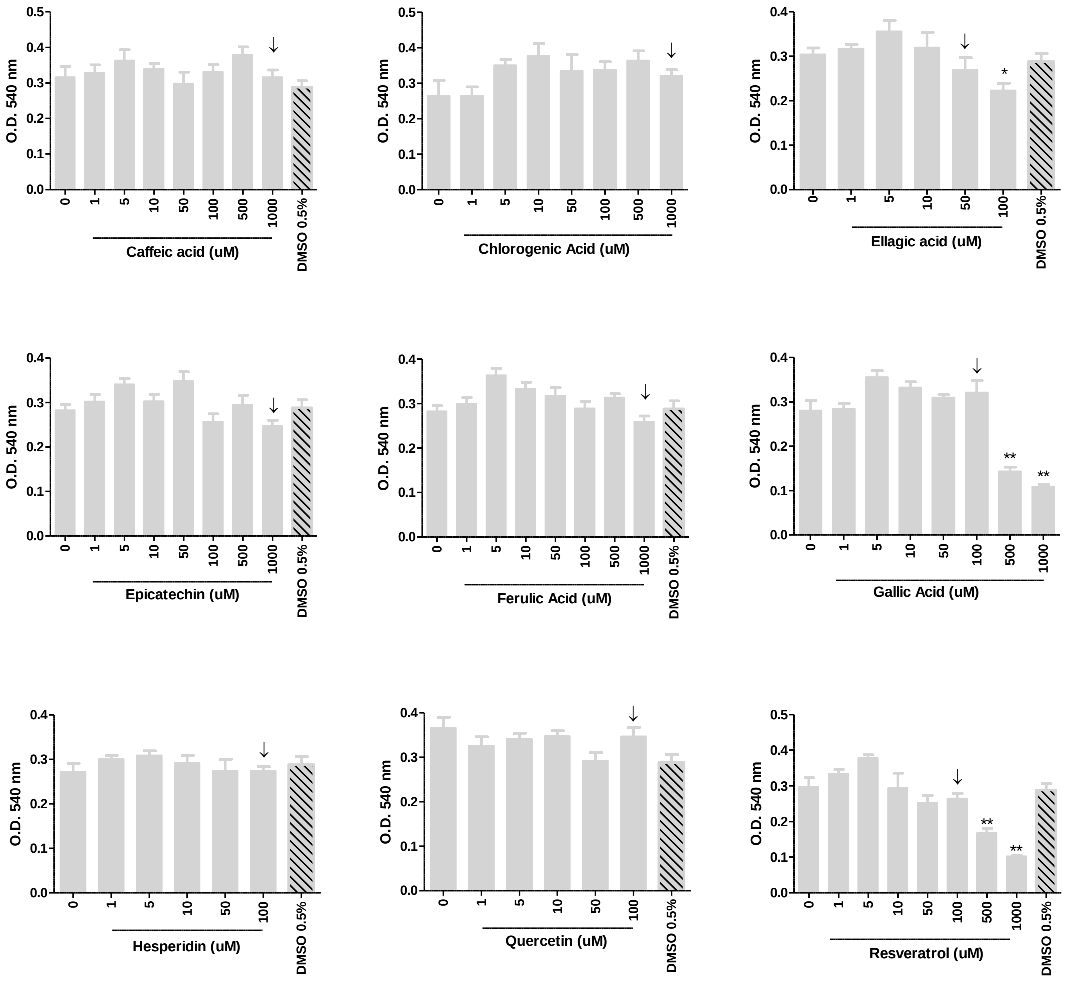

Figure S3: Cytotoxicity evaluated in control MRC-5 cells incubated during 24 h with a concentration range of the polyphenols caffeic acid (1-1000 µM), chlorogenic acid (1-1000 µM), ellagic acid (1-100 µM), epicatechin (1-1000 µM), ferulic acid (1-1000 µM), gallic acid (1-1000 µM), hesperidin (1-100 µM), quercetin (1-100 µM) and resveratrol (1-1000 µM). The experiment was carried out in triplicate with one repetition. ** $p<0.01$ and * $p<0.05$ when compared to non-treated MRC-5 cells. The arrows show the concentration chosen for each polyphenol for subsequent assays.

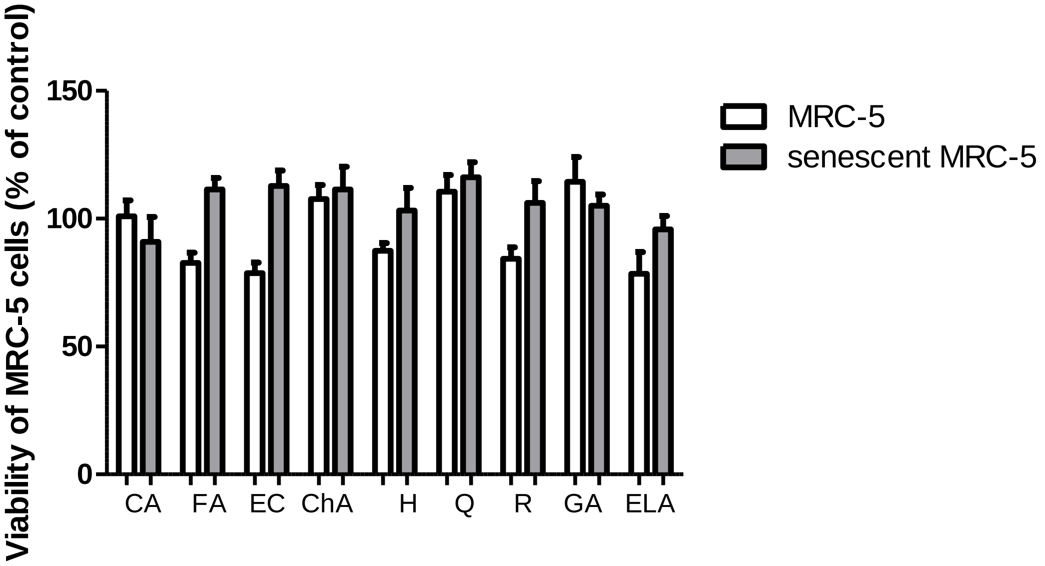

Figure S4: Cytotoxic activity in MRC-5 cells or senescent MRC-5 cells by isolated caffeic acid (CA, 1000 µM), chlorogenic acid (ChA, 1000 µM), ellagic acid (50 µM), epicatechin (EC, 1000 µM), ferulic acid (FA, 1000 µM), gallic acid (100 µM), hesperidin (100 µM), quercetin (100 µM) or resveratrol (100 µM). The experiments were carried out in triplicate with one repetition.

Table S1: Primers sequence

Gene	Forward	Reverse
CDKN2A	GAGCAGCATGGAGCCTTC	CGTAACTATTCGGTGCGTTG
CDKN1A	TCACTGTCTTGTACCCTTGTGC	GGCGTTTGGAGTGGTAGAAA
b-actin	CCAACCGCGAGAAGATGA	CCAGAGGCGTACAGGGATAG

Table S2: Viability of control and senescent MRC-5 after 24 h dasatinib, ellagic acid, quercetin and resveratrol isolated or in combination and percentage of viability reduction of senolytic combinations.

Drug	Control MRC-5 viability	Senescent MRC-5 viability	% reduction
Dasatinib	81.9±1.1	80.4±5.8	9.8
Ellagic acid	78.5±8.4	95.8±5.3	-
Quercetin	110.6±6.4	116.1±5.9	-
Resveratrol	84.3±4.5	106.2±8.5	-
Dasatinib + Ellagic Acid	107.8±8.9	46.7±6.7	43.3
Dasatinib + Quercetin	105.3±7.7	51.7±6.5	49.2
Dasatinib + Resveratrol	84.43±0.4	37.1±7.4	43.9
